# Comparative Effects of Some Medicinal Plants: *Anacardium occidentale, Eucalyptus globulus, Psidium guajava,* and *Xylopia aethiopica* Extracts in Alloxan-Induced Diabetic Male Wistar Albino Rats

**DOI:** 10.1155/2014/203051

**Published:** 2014-11-27

**Authors:** Victor Eshu Okpashi, Bayim Peter-Robins Bayim, Margaret Obi-Abang

**Affiliations:** ^1^Department of Biochemistry, University of Nigeria, Nsukka, Nigeria; ^2^Department of Chemical Sciences, Cross River University of Technology, Calabar, Nigeria

## Abstract

Insulin therapy and oral antidiabetic agents/drugs used in the treatment of diabetes mellitus have not sufficiently proven to control hyperlipidemia, which is commonly associated with the diabetes mellitus. Again the hopes that traditional medicine and natural plants seem to trigger researchers in this area is yet to be discovered. This research was designed to compare the biochemical effects of some medicinal plants in alloxan-induced diabetic male Wistar rats using named plants that are best at lowering blood glucose and hyperlipidemia and ameliorating other complications of diabetes mellitus by methods of combined therapy. The results obtained showed 82% decrease in blood glucose concentration after the 10th hour to the fortieth hour. There was significant increase *P* < 0.05 in the superoxide dismutase activity of the test group administered 100 mg/kg of *A. Occidentale*. There was no significant difference *P* > 0.05 recorded in the glutathione peroxidase activity of *E. globulus* (100 mg/kg) when compared to the test groups of *P. guajava* (250 mg/kg) and *X. aethiopica* (250 mg/kg). Catalase activity showed significant increase *P* < 0.05 in the catalase activity, compared to test groups. While at *P* > 0.05, there was no significant difference seen between test group and treated groups. Meanwhile, degree of significance was observed in other parameters analysed. The biochemical analysis conducted in this study showed positive result, attesting to facts from previous works. Though these individual plants extracts exhibited significant increase in amelorating diabetes complication and blood glucose control compared to glibenclamide, a synthetic antidiabetic drug. Greater performance was observed in the synergy groups. Therefore, a poly/combined formulation of these plants extracts yielded significant result as well as resolving some other complications associated with diabetics.

## 1. Introduction

We envisage that using only one or two of these plants extracts separately to determine its antidiabetic activities may not yield effective result. Therefore we set out to combine the plants extracts to investigate their effect arising from more than two entities that will produce an effect greater than their individual effects.

The effect of applying them synergistically to ameliorate diabetes mellitus and its associate diseases was mainly designed. Insulin therapy and oral antidiabetic agents/drugs used in the treatment of diabetes mellitus have not sufficiently proven to control hyperlipidemia. Also the adverse effect/limitations of both modes of treatments, together with the cost of procuring these drugs, have led to several research works on the efficiency of the alleged hypoglycemic and antidiabetic activity of these plants. Therefore this research work is geared towards the study of the named plants that are best at lowering blood glucose and hyperlipidemia and ameliorating other complications of diabetes mellitus.

**Figure 1:**
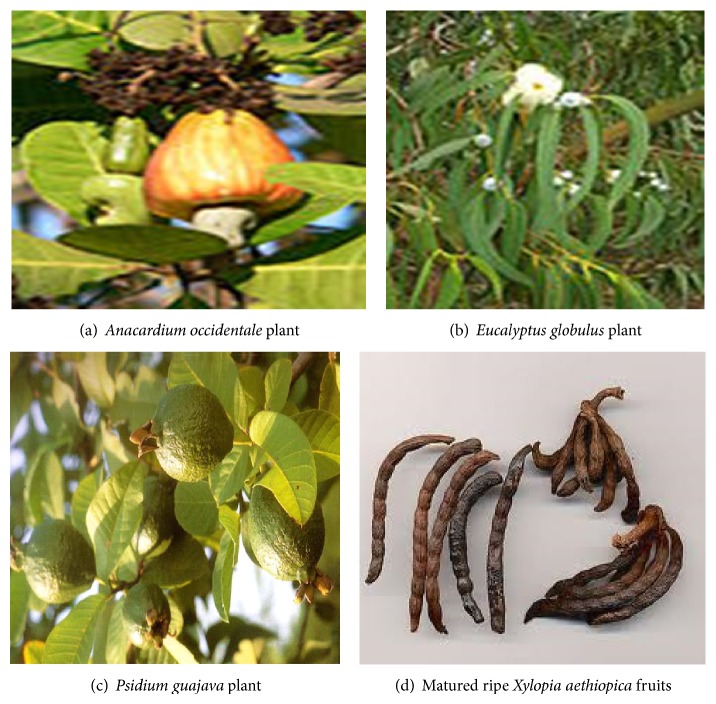
Medicinal plants used as combine therapy for this research.

*Anacardium occidentale L*. (see Figure 1(a)) leaves stem and bark extracts are utilized widely for the treatment of diarrhea, dysentery, and colonic pain. It has also been reported to possess antidiabetic, antibacterial, anti-inflammatory, and antiulcerogenic properties [1]. The leaves are also used in Brazil for eczema, psoriasis, scrofula, dyspepsia, genital problems, and venereal diseases, as well as for impotence, bronchitis, cough, intestinal colic, leishmaniasis, and syphilis-related skin disorders. The seed coat and the shell that remains after the extraction of the nut are used as fuel for burning purposes [2].

The use of plants in traditional medicine has been identified as a means of studying the potentiality of future medicines. In the year 2000, over 122 compounds used in conventional medicine were identified by researchers as derivatives of “ethnomedical” plants sources, with 80% of these compounds used for the same or similar traditional ethnomedical [3]. Medicinal plants possess curative properties with secondary metabolites that vary in chemical structures such as saponins, tannins, essential oils, alkaloids, and flavonoids to mention but few [4]. They proved to have contributed to the treatment of diseases such as HIV/AIDS, malaria, diabetes, sickle-cell, anaemia, mental disorders [5], and microbial infections [6, 7]. Iwu et al. [6] reported that the main benefits of using plants derived medicine are that they are considerably affordable and safer compared to synthetic options, thus providing intense therapeutic benefits and more affordable treatments.

The major chemical composition of* Eucalyptus globulus* (see Figure 1(b)) are *α*-pinene, *β*-pinene, terpenes, tannins, and so forth [8]. The blue gum flowers are considered as vital source of nectar and pollen greens for bees, thus yielding honey while their leaves are used for herbal tea and for therapeutic purposes [9]. Studied the antidiabetic effects of this plant in the pancreatic islets of diabetic rats. Upon the administration of aqueous* Eucalyptus globulus, *a reduction in weight loss and increase in water and food intake compared to the streptozotocin untreated diabetic rats administered with streptozotocin.* Psidium guajava* is an evergreen small tree or shrub whose origin is America [10–24]. Its commonly called in different dialects as follows: English, Guava; Hausa: Gwaaba; Yoruba: Guafa; and Igbo: Ugwoba. Deguchi and Miyazaki [25] studied the effect of aqueous* Psidium guajava* (guava) leaf extract (see Figure 1(c)) on alpha-glucosidase enzymes (that can digest carbohydrate). Results showed that the extracts were able to inhibit the in vitro activities of maltase, sucrase and alpha-amylase in a dose dependent perspective. In vivo studies showed that aqueous extracts of* Psidium guajava* leaves inhibit both sucrase and maltase activities in the intestinal mucosa of diabetic mice, with natural inhibition of mixed competitive and noncompetitive inhibition [26] while the antioxidant status was elevated in a dose dependent manner [27].* Xylopia aethiopica* is an evergreen aromatic tree that belongs to the* Annonaceae* plant family, as shown in Figure 1(d). The fruits are said to have consisted of *β*-pinene (19%), *γ*-terpinene (14.7%),* trans*-pinocarveol (8.6%), and P-cymene (7.3%) as essential oil [28]. The extracts of dried* Xylopia aethiopica *fruit were found to inhibit human cervical cancer cell lines C33A, inducing apoptosis and cell cycle arrest in C33A cells in a dose dependency [29]. Adaramoye et al. [30] study the effect of dried fruit extracts protection on Wistar albino rats from adverse effect of whole body radiation. He reported that a synergic treatment attenuated the adverse effects of irradiation on liver glutathione S-transferase (GSH), catalase activities after a week of exposure.

Diabetes insipidus is a metabolic disorder caused by deficiency of vasopressin (antidiuretic) a pituitary hormone that regulates reabsorption of water by the kidney. It is characterized by the production of large volume of diluted urine and constant thirst, although it is a rare kind of disease. According to [31], diabetes mellitus is a chronic metabolic disorder of carbohydrates, proteins, and fats occurring in the endocrine system [32, 33] due to absolute or relative deficiency of insulin secretion with/without varying degree of insulin resistance [34, 35]. Thus, a combined therapy of these plants extracts become necessary as a trial version for controlling and treating diabetes and associate diseases.

## 2. Methodology

### 2.1. Methods

The leaves of these plants, namely,* Anacardium occidentale*,* Eucalyptus globulus*,* Psidium guajava,* were collected from the premises of University of Nigeria, Nsukka, while the fruits of* Xylopia aethiopica *were purchased from a local market in Delta State.

## 3. Materials

### 3.1. Animals

The experimental animals used in this study were male Wistar albino rats obtained from the Faculty of Veterinary Medicine, University of Nigeria, Nsukka. Their age ranged between 12 and 14 weeks old, with average body weight 170–260 g.

### 3.2. Experimental Design

Sixty male Wistar rats were housed in separate cages, acclimatized for seven days. Thereafter, they were divided into fifteen groups of four rats per cage. They were maintained for 12 hours light and dark cycle at tropical conditions. The rats had free access to water and were fed with normal rat diet ad libitum. The feeds were obtained from Vital Feeds, UAC Nigeria. In the beginning of the experiment, the rats were weighted in order to predetermine dosage. They were grouped into various treatment groups from groups 1 to XV. However, groups 1-XIV were all diabetic; treatment was administered for 40 hours, arranged as follows.

 Group I was given* Anacardium occidentale* 100 mg/kg body weight, orally.
 Group II also was administered 250 mg/kg body weight of* Anacardium occidentale* orally.
 Group III received orally 100 mg/kg body weight* Eucalyptus globulus*.
 Group IV was given 250 mg/kg body weight of* Eucalyptus globulus* orally.
 Group V got orally 100 mg/kg body weight of* Psidium guajava*.
 Group VI also was administered orally 250 mg/kg body weight* Psidium guajava*.
 Group VII received 100 mg/kg body weight* Xylopia aethiopica* orally.
 Group VIII was given orally 250 mg/kg body weight* Xylopia aethiopica*.
 Group IX was administered a mixture of 100 mg/kg body weight* Anacardium occidentale* +* Eucalyptus globulus* orally.
 Group X received 250 mg/kg body weight of the mixture of* Anacardium occidentale* +* Eucalyptus globulus* by administration.
 Group XI was given the mixture,* Psidium guajava* +* Xylopia aethiopica* of 100 mg/kg body weight through oral route of administration.
 Group XII was administered 250 mg/kg body weight* Psidium guajava* +* Xylopia aethiopica* orally.
 Group XIII received 5 mg/kg body weight via oral intervention of glibenclamide.
 Group XIV diabetic but administered no treatment rather was fed with just feed and water.
 Group XV was given 10% dimethyl sulphoxide DMSO to act as positive control.

### 3.3. Chemicals/Reagents/Samples

Dimethyl sulphoxide (DMSO) was purchased from Serva, Heidelberg, New York, while chloroform (trichloromethane), sulphuric acid and alloxan monohydrate were bought from Sigma Aldrich Chemicals, Germany. Thiobarbituric acid was from Lab Tech chemical, Avishkar, and trichloroacetic acid from Kermel. Sodium chloride, cupric acid were purchase from Mayer and Baker, England. The commercial kits were of Randox laboratories Ltd, Crumlin Co. Antrim, UK.

## 4. Induction of Diabetes 

A quantity of 180 mg/kg alloxan monohydrate was dissolved in normal saline (0.9% Nacl) and administered intraperitoneally to induce diabetes, after subjecting the animals to fasting for 20 hours with their base lines blood glucose determined. Three days after induction of diabetes, the rats with fasting blood glucose higher than 250 mg/dL were used for the experiments. Blood glucose concentrations were determined every eight hours for 40 hours. Meanwhile, food and water were removed from cages four hours prior to testing. While the administration of the plant extracts was carried out every 12 hours. In the end of 40th hour, cardiac dislocation method was used to sacrifice the animals while blood and liver samples were collected for further biochemical test.

## 5. Preparation of Plant Extracts 

The leaves of three plants were air dried to constant weight at room temperature and reduced to powder by a commercial miller. Quantities of 600 g of each of plant such as* Anacardium occidentale* +* Eucalyptus globulus, Psidium guajava* +* Xylopia aethiopica* were pulverized, and plants materials were macerated in 2.7 litres of analytic chloroform. After 48 hours, the resulting extracts were filtered with Whatman number 1 filter paper and the filtrates were concentrated with rotary evaporator at minimal pressure; the yield of extracts was calculated using the following (see also Table 1):

(1) $$\begin{array}{c} \frac{\text{Mass}\;\text{of}\;\text{extract}\;\text{after}\;\text{rotary}\;\text{evaporation}}{\text{Mass}\;\text{of}\;\text{crude}\;\text{extra}}\times \frac{100}{1}. \end{array}$$

Quantity 8.3 g of each of the extracts were dissolved in 16 mL of 10% dimethyl sulphoxide (DMSO) and made up to 25 mL with distilled water; these were used as stock solution for the test. Each dose of the various extracts administered was calculated using (2), where the volumes given were arrived out as follows:

(2) VmL=D×PC,

where *D*: dose used (g/kg body weight of test animal), *P*: body weight (g), *C*: concentration (g/mL), and *V*: volume (mL).

## 6. Phytochemical Analysis 

The crude extracts of each plant were screened/analyzed qualitatively by the method modified by [36] for the presence of alkaloids, saponins, steroids, flavonoids, glycosides, tannin, and oils.

## 7. Statistical Analysis

Statistical analysis was conducted using SSPSS version 16, for the determination of mean values of the various biochemical parameters by method of one way analysis of variance.

## 8. Results

**Figure 2:**
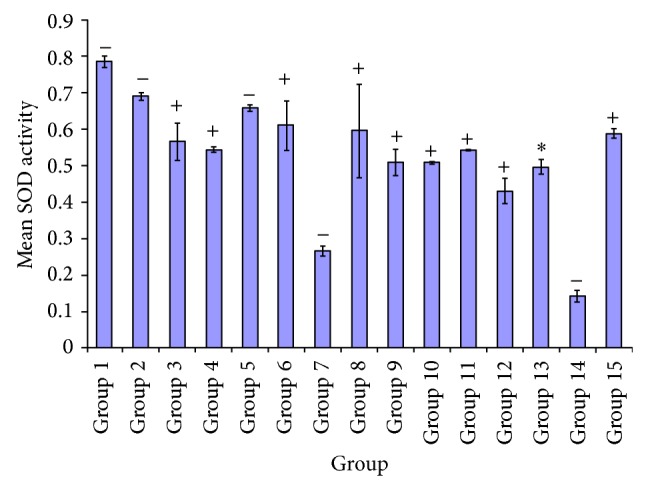
Comparative effects of some plants extracts in the superoxide dismutase activity.

Figure 2 indicates the superoxide dismutase activities of the experimental rats, showing the effect of various extracts at variable doses. The diabetic untreated group had the least activity and upon administration of these extracts culminated in appreciable increase in the SOD activity of the treated groups. Figure 2 also indicated significant *P* < 0.05 in superoxide dismutase activity of test group administered 100 mg/kg of* A. occidentale* compared to groups treated with* E. globulus *(100, 250 mg/kg),* P. guajava* (250 mg/kg),* X. aethiopica* (100, 250 mg/kg),* A. occidentale* +* E. globulus *(100, 250 mg/kg),* P. guajava* +* X. aethiopica* (100, 250 mg/kg), and glibenclamide (5 mg/kg), respectively. Meanwhile no significant difference *P* > 0.05 was observed in groups administered with* A. occidentale* (250 mg/kg) and* P. guajava* (100 mg/kg). A significant *P* < 0.05 was found between 250 mg/kg,* A. occidentale* treated group, and test groups* E. globulus* (250 mg/kg),* X. aethiopica *(100 mg/kg),* A. occidentale* +* E. globulus *(100, 250 mg/kg),* P. guajava* +* X. aethiopica* (100, 250 mg/kg), glibenclamide (5 mg/kg), and diabetic untreated. There was no significant difference *P* > 0.05 in the* A. occidentale* 250 mg/kg compared to groups* A. occidentale* (100 mg/kg),* E. globulus* (100 mg/kg),* P. guajava* (100, 250 mg/kg),* X. aethiopica*, and DMSO control.

In groups 9 and 14, superoxide dismutase activity in test group showed significant increased *P* < 0.05 compared to treated groups 1, 2, 5, 7, and 14. While with groups 3, 4, 6, 8, 10, 11, 12, 13, and 15 they were not significantly different *P* > 0.05, from group 9. Likewise group 10, there was a significant increase *P* < 0.05 indicated between groups 1, 2, 5, 7, and 14.

**Figure 3:**
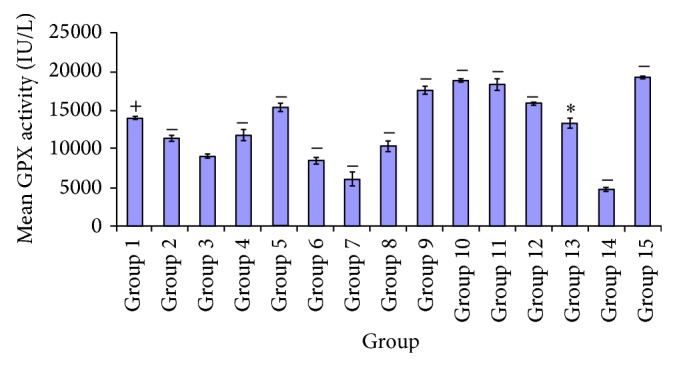
Comparative effect of the various plant extracts on glutathione peroxidase activity.

In Figure 3 a significant increase *P* < 0.05 was observed in groups administered 100 mg/kg of* A. Occidentale*, 250 mg/kg,* E. globulus* (100, 250 mg/kg),* P. guajava* (250 mg/kg),* X. aethiopica* (100, 250 mg/kg),* A. occidentale* +* E. globulus* (100, 250 mg/kg), and* P. guajava* +* X. aethiopica* (100, 250 mg/kg), and* P. guajava* +* X. aethiopica* (100, 50 mg/kg), and DMSO control group. Considering these, there was no significant difference *P* > 0.05 observed in groups given* P. guajava* (100 mg/kg) and glibenclamide (5 mg/kg). As shown in groups 1, 2, and 3, a significant increase *P* < 0.05 was recorded in groups 1 to 15, respectively. Except for group 2, 4 and 8 showed no significant difference *P* > 0.05. A significant increase *P* < 0.05 was observed in group 4, in the test group administered 100 mg/kg of* E. globulus* and the treatment groups of* A. occidentale* (100, 250 mg/kg),* E. globulus* (250 mg/kg),* P. guajava* (100 mg/kg),* X. aethiopica* (100 mg/kg),* A. occidentale* +* E. globulus* (100, 250 mg/kg),* P. guajava + X. aethiopica *(100, 250 mg/kg), and glibenclamide (5 mg/kg). There was no significant difference *P* > 0.05 recorded in the glutathione peroxidase activity of* E. globulus* (100 mg/kg) compared to the test groups of* P. guajava* (250 mg/kg) and* X. aethiopica* (250 mg/kg). At *P* < 0.05, a significant increase occurred between test group 4 and treatment groups 1, 3, 5, 6, 7, 9, 10, 11, 12, 13, 14, and 15. No significant difference *P* > 0.05 was shown between group 4 and groups 2 and 8.

Group 15 indicated a significant increase *P* < 0.05 between test groups 2, 3, 4, 6, 7, 8, 9, 10, 11, 13, and 14 compared to group 5. However, there were no significant differences *P* > 0.05 between test group 5 and treatment groups 1 and 12. Meanwhile in group 6 the glutathione peroxidase activity was found to be significantly different *P* < 0.05 compared to groups 1, 2, 4, 5, 7, 8, 9, 10, 11, 12, 13, 14, and 15. There was no significant difference *P* > 0.05 found in groups 6 and 3. As shown in group 2, there were significant differences *P* < 0.05 between the test group of* X. aethiopica *(100 mg/kg) and the treatment groups of* A. occidentale* (100, 250 mg/kg),* E. globulus *(100, 250 mg/kg),* P. guajava* (100, 250 mg/kg),* X. aethiopica* (250 mg/kg),* A. occidentale *+* E. globulus* (100, 250 mg/kg),* P. guajava* +* X. aethiopica* (100, 250 mg/kg), glibenclamide and DMSO. No significant difference *P* > 0.05 was observed in the treatment group of 100 mg/kg* X. aethiopica* compared to the diabetic untreated group. In the 250 mg/kg of* X. aethiopica* group there was a significant difference *P* < 0.05 shown to have taken place among the test groups* A. occidentale* (100 mg/kg),* P. guajava* (100, 250 mg/kg),* X. aethiopica* (100 mg/kg),* P. guajava* (100, 250 mg/kg),* X. aethiopica* (100 mg/kg),* A. occidentale* +* E. globulus* (100, 250 mg/kg),* P. guajava *+* X. aethiopica* (100, 250 mg/kg), and glibenclamide (5 mg/kg). There were no significant differences *P* > 0.05 observed in groups* A. occidentale *(250 mg/kg) and* E. globulus* (100, 250 mg/kg). From group 4 a significant difference *P* < 0.05 was observed in* A. occidentale + E. globulus* (100 mg/kg) test group, when compared with the treatment group of* A. occidentale* (100, 250 mg/kg),* E. globulus* (100, 250 mg/kg),* P. guajava* (100, 250 mg/kg),* X. aethiopica *(100, 250 mg/kg),* P. guajava* +* X. aethiopica* (250 mg/kg), and 5 mg/kg of glibenclamide. However no significant difference *P* > 0.05 was indicated between* A. occidentale* +* E. globulus* (100 mg/kg) and groups* A. occidentale + E. globulus* (250 mg/k) and* P. guajava* +* X. aethiopica* (100 mg/kg). In the* A. occidentale* +* E. globulus* 250 mg/kg group a significant increase *P* < 0.05 was observed, compared with the test groups of* A. occidentale* (100, 250 mg/kg),* E. globulus* (100, 250 mg/kg),* P. guajava *(100, 250 mg/kg),* X. aethiopica* (100, 250 mg/kg),* P. guajava* +* X. aethiopica* (250 mg/kg), glibenclamide (5 mg/kg), and diabetic untreated group while at *P* > 0.05 no significant difference was seen between these treatment groups and* A. occidentale* +* E. globulus* (100 mg/kg),* P. guajava* +* X. aethiopica *(100 mg/kg), and DMSO control groups.

There were significant differences *P* < 0.05 observed in group 11 when compared to test groups 1, 2, 3, 4, 5, 6, 7, 8, 12, 13, and 14. No significant difference *P* > 0.05 was indicated between group 11 and groups 9, 10, and 15. Moreover group 12 was recorded to be significantly different at *P* < 0.05 as shown in group 15, the glutathione peroxidase activity of this group as compared with test groups 1, 2, 3, 4, 6, 7, 8, 9, 10, 11, 13, and 14. Also group 12 showed no significant difference *P* > 0.05.

A significant difference *P* < 0.05 was indicated in group 4; the group administered 100 mg/kg of* A. occidentale* was compared to* E. globulus* (250 mg/kg),* P. guajava* (100, 250 mg/kg),* X. aethiopica* (100 mg/kg),* A. occidentale* +* E. globulus* (100, 250 mg/kg),* P. guajava* +* X. aethiopica *(100, 250 mg/kg), 5 mg/kg of glibenclamide, and diabetic untreated group while there were no significant differences *P* > 0.05 observed in groups given* A. occidentale* (250 mg/kg),* E. globulus* (100 mg/kg),* X. aethiopica* (250 mg/kg), and DMSO control. The 250 mg/kg* A. occidentale *test showed significant decrease *P* < 0.05 compared with the diabetic untreated group. Other groups show no significant difference *P* > 0.05. Meanwhile, it was observed that a significant decrease *P* < 0.05 occurred between group treated with 250 mg/kg of* E. Globulus*, test groups* A. occidentale* (100 mg/kg),* E. globulus* (100 mg/kg), and diabetic untreated group while no significant difference *P* > 0.05 was indicated between the test groups* E. globulus* (250 mg/kg) treated groups* A. occidentale* (250 mg/kg),* A. occidentale* +* E. globulus* (100, 250 mg/kg),* P. guajava* +* X. aethiopica *(100, 250 mg/kg), glibenclamide (5 mg/kg), and DMSO.

In group 2* X. aethiopica* (100 mg/kg) there was a significant difference *P* < 0.05 compared to test groups* A. occidentale* (100 mg/kg),* E. globulus *(100 mg/kg), and diabetic untreated group. No significant difference *P* > 0.05 was observed in treated groups* A. occidentale* (250 mg/kg),* E. globulus* (250 mg/kg),* P. guajava* (100, 250 mg/kg),* X. aethiopica* (250 mg/kg),* A. occidentale *+* E. globulus* (100, 250 mg/kg),* P. guajava* +* X. aethiopica* (100, 250 mg/kg), glibenclamide, and DMSO. A significant difference *P* < 0.05 was observed in the groups administered 250 mg/kg of* X. aethiopica* and diabetic untreated group while the groups administered with* A. occidentale* (100, 250 mg/kg),* E. globulus* (100, 250 mg/kg),* P. guajava* (100, 250 mg/kg),* X. aethiopica* (100, 250 mg/kg),* A. occidentale* +* E. globulus* (100, 250 mg/kg),* Psidium *+* X. aethiopica* (100, 250 mg/kg), and 5 mg/kg glibenclamide show significant difference *P* < 0.05 compared to the* X. aethiopica* (250 mg/kg). There was significant difference *P* < 0.05 observed in group 9 compared to groups 1, 3, and 14. However no significant difference was indicated between groups 2, 4, 5, 6, 7, 8, 9, 10, 11, 12, 13, and 14. Though group 10 show significant decrease *P* < 0.05 compared to groups 1, 3, and, 14 and no significant difference *P* > 0.05 was observed among groups 2, 4, 5, 6, 7, 8, 9, 11, 12, 13, and 15.

The 5 mg/kg of glibenclamide test group was found to be significantly different *P* < 0.05, compared to groups* A. occidentale* (100 mg/kg),* E. globulus* (100 mg/kg) and diabetic untreated group.

Figure 5 shows the effect of some variable doses of each extract on triglyceride concentrations of experimental rats, with diabetic untreated group having the highest triglyceride value. In other groups, apart from DMSO, upon administration of these extracts, subsequent decrease in various treatment groups especially 250 mg/kg was seen. Group 7 showed significant decrease *P* < 0.05 in triglyceride level of test group* A. occidentale* 250 mg/kg and treatment groups given* P. guajava* (100 mg/kg),* X. aethiopica* (100 mg/kg), and 5 mg/kg glibenclamide, and diabetic untreated group. However no significant difference *P* > 0.05 was observed in groups* A. occidentale* (100 mg/kg),* E. globulus* (100, 250 mg/kg),* P. guajava* (250 mg/kg),* X. aethiopica* (100, 250 mg/kg),* P. guajava* +* X. aethiopica* (100, 250 mg/kg), and DMSO control. A significant difference *P* < 0.05 was observed in group 5, compared to test groups 2, 10, 11, 12, 14, and 15.

A significant difference *P* < 0.05 occurred at the test group administered 100 mg/kg* X. aethiopica* and treated groups* A. occidentale* (250 mg/kg),* E. globulus *(250 mg/kg),* X. aethiopica* (250 mg/kg),* A. occidentale* +* E. globulus* (100 mg/kg), and diabetic untreated and DMSO control groups. No significant difference *P* > 0.05 was shown in groups* A. occidentale* (100 mg/kg),* E. globulus* (100 mg/kg),* P. guajava* (100, 250 mg/kg),* A. occidentale* +* E. globulus* (250 mg/kg), and glibenclamide (5 mg/kg). Nevertheless a significant decrease *P* < 0.05 was recorded in* X. aethiopica* 250 mg/kg test group compared to treated groups* X. aethiopica* (100 mg/kg) and 5 mg/kg of glibenclamide and diabetic untreated group. There was no significant difference *P* > 0.05 in test groups* A. occidentale* (100, 250 mg/kg),* E. globulus* (100, 250 mg/kg),* P. guajava* (100, 250 mg/kg),* A. occidentale* +* E. globulus *(100, 250 mg/kg),* P. guajava* +* X. aethiopica* (100, 250 mg/kg), and DMSO.

Figure 6 shows the effect of variable extracts on aspartate aminotransferase activity, at varying doses in experimental rats where the diabetic untreated group had the highest activity/performance of this enzyme. Upon treatment with this extracts led to successive reduction in AST level in the treated groups. Group 6 showed significant difference *P* < 0.05, from groups 1, 4, 5, 6, 9, 10, 11, 12, 14, and 15. No significant difference was shown *P* > 0.05 in groups 2, 3, 7, 8, and 13 but, in group 6, there was a significant decrease *P* < 0.05 indicated in test groups administered with 250 mg/kg of* A. occidentale* when compared with the treated groups administered with* P. guajava *(250 mg/kg) and* A. occidentale* +* E. globulus* (250 mg/kg). No significant difference *P* > 0.05 was observed in the test group given 250 mg/kg* A. occidentale* and the treated groups administered* A. occidentale *(100 mg/kg),* E. globulus* (100, 250 mg/kg),* P. guajava* (100 mg/kg),* X. aethiopica* (100, 250 mg/kg),* P*.* guajava* +* X*.* aethiopica *(250 mg/kg), and 5 mg/kg of glibenclamide. In group 6, a significant decrease was observed *P* < 0.05 in its aspartate aminotransferase status compared to groups 1, 2, 3, 4, 7, 8, 12, 13, and 14, as shown in group 6. No significant difference *P* > 0.05 was indicated from groups 5, 6, 9, 10, 11, and 15. From group 6, a significant difference *P* < 0.05 was recorded between test group* X. aethiopica* 100 mg/kg and the treatment groups of* P. guajava* (100, 250 mg/kg),* A. occidentale* +* E. globulus* (100, 250 mg/kg),* P. guajava* +* X. aethiopica* (100 mg/kg), diabetic untreated group, and glibenclamide (5 mg/kg). There was no significant difference *P* > 0.05 shown in aspartate aminotransferase state of the group administered 100 mg/kg of* X. aethiopica* compared to the test groups of* A. occidentale* (100, 250 mg/kg),* E. globulus* (100, 250 mg/kg),* X. aethiopica* (250 mg/kg),* P. guajava* +* E. globulus* (250 mg/kg), and glibenclamide (5 mg/kg). Group 6 showed significant decrease between group 10 and test groups 1, 2, 3, 4, 5, 7, 8, 9, 11, 12, 13, and 14, but no significant difference *P* > 0.05 was observed between group 10 and treatment groups 6, 11, and 15. In the same group 6, there was a significant difference *P* < 0.05 in treated groups administered with* P. guajava* +* X. aethiopica* (100 mg/kg) and test groups of* A. occidentale* (100, 250 mg/kg),* E. globulus* (100, 250 mg/kg),* X. aethiopica* (100, 250 mg/kg),* P. guajava* (250 mg/kg), and glibenclamide (5 mg/kg), and diabetic untreated group.

Results of ALT level which is unusually associated with diabetic untreated group were very high. Hence treatment with combined extracts culminated in progressive decrease in ALT levels, depicted in the treated groups. There was significant difference *P* < 0.05 observed in test group administered 100 mg/kg of* A. occidentale*, compared with treated groups given* X. aethiopica* (100, 250 mg/kg),* A. occidentale* +* E. globulus* (100, 250 mg/kg), and* P. guajava* +* X. aethiopica* (100, 250 mg/kg), and DMSO control. There was significant decrease *P* < 0.05 shown in group 7, between the test group 2 and treated groups given 8, 9, 10, 11, 12, 14, and 15. However no significant difference *P* > 0.05 was observed in group 2 and the test groups 1, 3, 4, 5, 6, 7, and 13, respectively. A significant difference *P* < 0.05 occurred at test group 4 and treated groups 8, 9, 10, 11, 12, 14, and 15 as shown in Figure 7. While no significant difference *P* > 0.05 was observed in group 4, compared to treated groups 1, 2, 3, 5, 6, 7, and 13. There was significant difference *P* < 0.05 found in the test group given 100 mg/kg* P. guajava* and groups given* A. occidentale* +* E. globulus* (100, 250 mg/kg), and* P. guajava* +* X. aethiopica* (100, 250 mg/kg). No significant difference was noticed *P* > 0.05 in the alanine aminotransferase activity of* P. guajava* 100 mg/kg group and* A. occidentale* (100, 250 mg/kg),* E. globulus* (100, 250 mg/kg),* P. guajava* (250 mg/kg),* X. aethiopica* (100, 250 mg/kg), and glibenclamide (5 mg/kg). There was a significant decrease *P* < 0.05 observed in alanine aminotransferase level in test group 8, compared to treated groups 1, 2, 3, 4, 11, and 14. No significant difference *P* > 0.05 was indicated in groups 5, 6, 7, 8, 9, 10, 12, 13, and 14. Test group 10 showed significant decrease *P* < 0.05, compared to groups 1, 2, 3, 4, 5, 6, 7, 13, and 14, respectively. There was no significant difference *P* > 0.05 observed in group 10 and groups 8, 9, 11, 12, and 15. *P* < 0.05. Significant decrease was indicated in the test group administered* P. guajava* +* X. aethiopica* (100 mg/kg) and the treated groups administered* A. occidentale* (100, 250 mg/kg),* X. aethiopica* (100, 250 mg/kg),* E. globulus* (100, 250 mg/kg),* P. guajava* (100, 250 mg/kg), and glibenclamide (5 mg/kg), and diabetic untreated group. However no significant difference between the test group* P. guajava* +* X. aethiopica* (100 mg/kg) and the treated groups of* A. occidentale* +* E. globulus* (100, 250 mg/kg) and DMSO control.

Figure 8 showed significant decrease *P* < 0.05 in alkaline phosphatase activity in the test group administered 100 mg/kg body weight* A. occidentale*, compared to the test groups treated with* A. occidentale* (250 mg/kg),* E. globulus* (100, 250 mg/kg),* X. aethiopica* (250 mg/kg), and glibenclamide (5 mg/kg). There was no significant difference *P* > 0.05 observed in group treated with* A. occidentale* (100 mg/kg) and the test groups given* P. guajava* (250 mg/kg),* X. aethiopica* (100 mg/kg),* A. occidentale* +* E. globulus* (100, 250 mg/kg),* P. guajava* +* E. globulus* (100, 250 mg/kg), and DMSO control groups. A significant difference *P* < 0.05 was seen in groups 3 and 5 between test group 2 and all other test groups. A significant difference *P* < 0.05 was observed in Figure 8 between the test group 8 and groups 1, 2, 3, 5, 6, 7, 9, 10, 14, and 15. Moreover no significant difference *P* > 0.05 was indicated in test group 4, 8, and 15. In Figure 8, the significant difference was recorded in the treatment group administered 100 mg/kg* A. occidentale* +* E. globulus* and the test groups given* A. occidentale* (250 mg/kg),* E.* globulus (100, 250 mg/kg),* P. guajava* (100 mg/kg),* X. aethiopica* (100 mg/kg), glibenclamide (5 mg/kg),* X. aethiopica* (100 mg/kg), and glibenclamide (5 mg/kg), and diabetic untreated group. Nevertheless no significant difference *P* > 0.05 was indicated among test group administered* A. occidentale* +* E. globulus* (100, 250 mg/kg),* P. guajava* (250 mg/kg),* X. aethiopica* (100 mg/kg),* P. guajava* +* X. aethiopica* (100, 250 mg/kg),* A. occidentale* +* E. globulus *(250 mg/kg), and DMSO control.

Figure 8 shows significant increase *P* < 0.05 in alkaline phosphatase activity between group 14 and other groups. Also there was a significant difference *P* < 0.05 observed between the DMSO control group and the test groups administered* A. occidentale* (250 mg/kg),* E. globulus* (100, 250 mg/kg),* P. guajava *(100 mg/kg),* X. aethiopica* (250 mg/kg),* P. guajava* +* X. aethiopica* (250 mg/kg), and glibenclamide (5 mg/kg), and diabetic untreated group.

Diabetes mellitus is characterised by lipid peroxidation of membrane organelle, with MDA as index of this biochemical process. Thus on event of diabetes, if poorly managed there is increase in MDA concentration as shown in the diabetic untreated group in Figure 9. However the administration of synergetic extracts resulted in a simultaneous decrease in MDA concentration, as seen in the treated groups. A significant decrease was observed *P* < 0.05 in Figure 9, between test group* A. occidentale* (100 mg/kg) and treatment groups* A. occidentale* (250 mg/kg),* E. globulus* (250 mg/kg),* P. guajava* (100, 250 mg/kg),* X. aethiopica* (100 mg/kg),* A. occidentale* +* E. globulus* (100, 250 mg/kg), and glibenclamide (5 mg/kg), diabetic untreated group, and DMSO group while no significant difference *P* > 0.05 was indicated in groups given* A. occidentale* (100 mg/kg),* X. aethiopica* (250 mg/kg), and* P. guajava* +* X. aethiopica* (100, 250 mg/kg). Also there was significant difference *P* < 0.05 seen in the test group administered* A. occidentale *250 mg/kg compared to treatment groups of* A. occidentale *(100 mg/kg),* E. globulus* (250 mg/kg),* X. aethiopica* (250 mg/kg),* A*.* occidentale* +* E. globulus* (250 mg/kg), and* P. guajava* +* X. aethiopica* (100, 250 mg/kg). No significant difference at *P* > 0.05 was recorded between* A. occidentale* (250 mg/kg) and the groups administered with* E. globulus* (100 mg/kg),* P. guajava* (100, 250 mg/kg),* X. aethiopica* (100 mg/kg),* A. occidentale* +* E. globulus* (100 mg/kg), and glibenclamide (5 mg/kg). Figure 9 showed significant difference *P* < 0.05 between test group administered 100 mg/kg* X. aethiopica* and treatment groups of* A. occidentale* (100 mg/kg),* E. globulus* (100, 250 mg/kg),* P. guajava* (100, 250 mg/kg),* X. aethiopica* (250 mg/kg),* A. occidentale* +* E. globulus* (100, 250 mg/kg),* P. guajava* +* X. aethiopica *(100, 250 mg/kg), and 5 mg/kg of glibenclamide. Regardless of this, no significant difference *P* > 0.05 was observed in group given* A*.* occidentale* (250 mg/kg). With 250 mg/kg of* X. aethiopica* test group, there was significant difference *P* < 0.05 recorded between this group and treated groups of* A. occidentale* (250 mg/kg),* E. globulus *(100, 250 mg/kg),* P. guajava* (100, 250 mg/kg),* X. aethiopica* (100 mg/kg),* A. occidentale* +* E. globulus* (100, 250 mg/kg), and glibenclamide (5 mg/kg). No significant difference *P* > 0.05 was observed in test groups of* A. occidentale* (100 mg/kg) and* P. guajava* +* X. aethiopica* (100, 250 mg/kg).

Figure 10 depicts the effect of diabetes on antioxidant catalase, where diabetic untreated group showed low enzyme activity after administration of various plants extracts culminated in significant elevations in treated groups, particularly in 250 mg/kg dose. This showed significant difference *P* < 0.05 in test group administered 100 mg/kg of* A. occidentale,* compared to treatment groups of* P. guajava *(250 mg/kg) and* P. guajava + X. aethiopica* (100, 250 mg/kg). There was no significant difference *P* > 0.05 indicated in groups administered* A. occidentale* (250 mg/kg),* E. globulus* (100, 250 mg/kg),* P. guajava* (100 mg/kg),* X. aethiopica* (100 mg/kg),* A. occidentale + E. globulus *(100, 250 mg/kg), and glibenclamide (5 mg/kg). A significant increase *P* < 0.05 was observed in the catalase activity of test group 2, as compared to treated groups 4, 11, 12, 14, and 15. No significant difference *P* > 0.05 was seen in group 2 and groups 1, 3, 5, 6, 7, 8, 9, 10, and 13. There was a significant difference *P* < 0.05 indicated in groups 4, 7, 14, and 15. Moreover, there was no significant difference *P* > 0.05 observed between test group 7 and test groups 1, 2, 3, 5, 6, 8, 9, 10, 11, 12, and 13. Whilst in group 8, a significant increase was observed *P* < 0.05 in the catalase activity, compared to other test groups 1, 3, 4, 9, 13, 14, and 15. Catalase activity of the treated group administered with 100 mg/kg of* A. occidentale* +* E. globulus* was shown to be significantly different *P* < 0.05 in groups administered with* P. guajava *(250 mg/kg),* X. aethiopica* (250 mg/kg), and* P. guajava* +* X. aethiopica* (100, 250 mg/kg). However there was no significant difference of this test group compared to treated groups administered with* A. occidentale* (100, 250 mg/kg),* E. globulus* (100, 250 mg/kg),* P. guajava* (100 mg/kg),* X. aethiopica* (100 mg/kg),* A. occidentale* +* E. globulus *(250 mg/kg), and glibenclamide (5 mg/kg). In group 13 *P* < 0.05 significant difference was indicated in the catalase activity, compared to test groups 6, 8, 11, 12, 14, and 15. Thus no significant difference *P* > 0.05 was observed in groups 1, 2, 3, 4, 5, 7, 9, 10, and 13 as shown in Figure 10.

Figure 11 indicated a significant increase in antioxidant vitamin C, upon administration of various extracts on event of diabetes, resulting in decrease concentration of vitamin C after administration of* A. occidentale*, 250 mg/kg dose. Significant increase was noticeable *P* < 0.05. Thus significant increase in the test group administered 100 mg/kg of* A. occidentale *was shown, as compared to the groups that received* A. occidentale* (250 mg/kg),* E. globulus* (100, 250 mg/kg),* P. guajava* (250 mg/kg), and* X. aethiopica* (100 mg/kg). In Figure 11, there was significant difference *P* < 0.05 observed amongst groups that received* A. occidentale* (250 mg/kg),* E. globulus* (100 mg/kg),* X. aethiopica *(100 mg/kg),* A. occidentale* +* E. globulus* (100 mg/kg),* P. guajava* +* X. aethiopica* (100 mg/kg), and glibenclamide (5 mg/kg), compared to test group* P. guajava* (100 mg/kg). No significant difference *P* > 0.05 was indicated in groups* A. occidentale* (100 mg/kg),* E. globulus* (250 mg/kg),* P. guajava *(250 mg/kg),* X. aethiopica* (250 mg/kg),* A. occidentale* +* E. globulus* (250 mg/kg), and* P. guajava* +* X. aethiopica* (250 mg/kg). Between test group 6 and treatment groups 1, 2, 3, 7, 9, 10, 11, 13, and 14 there was significant increase *P* < 0.05 recorded. There were no significant differences *P* > 0.05 observed in groups 4, 5, 8, 12, and 15. Significantly there was a difference *P* < 0.05 observed in groups that received 100 mg/kg* P. guajava* +* X. aethiopica* (100 mg/kg) and treatment groups* A. occidentale* (250 mg/kg),* E. globulus* (100, 250 mg/kg),* P. guajava* (100, 250 mg/kg),* X. aethiopica* (100 mg/kg), and* P. guajava* +* X. aethiopica* (250 mg/kg) while no significant difference *P* > 0.05 was shown in groups given* A. occidentale* (100 mg/kg),* X. aethiopica* (100, 250 mg/kg),* A. occidentale* +* E. globulus* (100, 250 mg/kg), and 5 mg/kg of glibenclamide. In test group 12, a significant increase *P* < 0.05 was shown with regard to the vitamin C concentration compared to groups 2, 3, 7, 9, 11, 13, 14, and 15. There were no significant differences *P* > 0.05 recorded in groups 1, 4, 5, 6, 8, and 10.

The results of blood glucose level of rats treated with 100 mg/kg body weight given* Anacardium occidentale* was more effective than glibenclamide, DMSO before the 10th hour. After this time there was 74% decrease in blood glucose up to the 40th hour compared to the diabetic untreated group. The group administered with 250 mg/kg of the same extracts, 70% reduction glucose concentration, was recorded at the 10th hour up to 40th hour. Group 2 showed 82% decrease in blood glucose concentration after the 10th hour to fortieth hour in the group given 100 mg/kg of* Eucalyptus globulus*, compared to 6.9% decrease in diabetic untreated group and 74% glibenclamide group. Thus, 60% reduction in blood glucose was observed in the group treated with 250 mg/kg of the same extracts. Blood glucose concentration reduced by 23% in the group given 100 mg/kg body weight of* Xylopia aethiopica* as shown in group 2, from the tenth hour to the fortieth hour. Considerable decrease in blood glucose of 82% occurred with 250 mg/kg in the group administered the same extracts from the tenth hour up to the fortieth hour, compared to the 6.9%, 74% of the diabetic untreated and glibenclamide groups while in the glibenclamide treated group 74% decline in glucose concentration from the tenth hour to the fortieth hour was shown compared to the 74% of* A. occidentale* 100, 250 mg/kg, 82, 60% of* E. globulus* of 100, 250 mg/kg, 50%* P. guajava* of 100, 250 mg/kg, and 23, 82% of* X. aethiopica* 100, 250 mg/kg body weight.

Figure 13 showed 83% decline in the test groups treated with 100 mg/kg body weight of* A. occidentale + E. globulus* while 79% reduction occurred in 250 mg/kg of the same extracts compared to 6.9% in blood glucose concentrations of diabetic untreated group and the 74% of glibenclamide group. The blood glucose concentrations in Figure 13 show 66% reduction in groups administered 100 mg/kg body weight of* P. guajava* +* X. aethiopica*, when compared to 6.9%, 74% of diabetic and glibenclamide treated groups. A 58% decrease was recorded for the group given 250 mg/kg of the same extracts, compared to the fall in glucose levels of diabetic untreated and glibenclamide groups while diabetic untreated group with 6.9% decline was observed in groups 12 and 13 concerning the blood glucose concentration, when compared to other test groups administered various extracts at tenth hour up to the fortieth hour.

## 9. Discussion 

Several parameters such as plasma, liver homogenates of the experimental animals from the various groups were analysed. These parameters so determined are indicators of diabetes and its associated complications. Some diabetic indicators are plasma blood glucose, blood lipids: cholesterol (CHO), triglyceride (TG), antioxidant: superoxide dismutase (SOD), glutathione peroxidase (GPX), catalase (CAT), and vitamin C. lipid peroxidation index malondialdehyde (MDA), liver marker enzymes: alanine aminotransferase (ALT), aspartate aminotransferase (AST), and alkaline phosphatase (ALP).

Figure 2 depicts the effect of the plant extracts at variable doses on superoxide dismutase activity. A more significant elevation in superoxides dismutase level in group administered* A. occidentale* (100, 250 mg/kg),* P. guajava* (100, 250 mg/kg), and* X. aethiopica* (250 mg/kg) while minimal increase was observed in treatment groups* E. globulus* (100, 250 mg/kg),* A. occidentale* +* E. globulus* (100, 250 mg/kg), and* P. guajava *+* X. aethiopica* (100 mg/kg) than glibenclamide treated group.

The activity of glutathione peroxidase is estimated from the plots of Figure 3, which shows greater increase in groups administered* A. occidentale* +* E. globulus* (100, 250 mg/kg),* P. guajava* +* X. aethiopica* (100, 250 mg/kg), and* P. guajava* (100 mg/kg) while a significant elevation was seen in* A. occidentale *(100 mg/kg) and* P. guajava *(100 mg/kg), compared to glibenclamide treated group.

**Figure 4:**
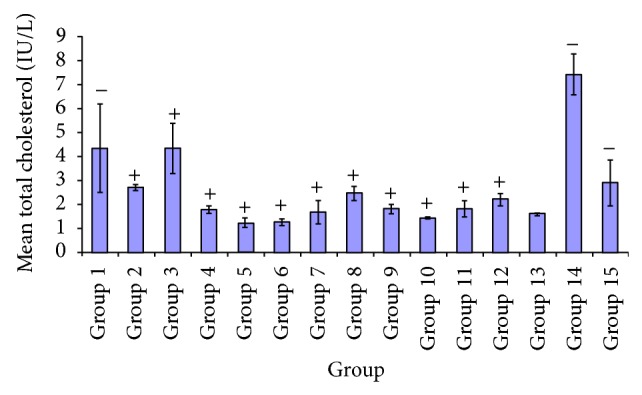
Comparative effect of some plants extracts on total cholesterol level.

Diabetes mellitus is characterized by distorted lipid metabolism, with increase in blood lipids. Hence Figure 4 showed the effect of varying extracts dose on cholesterol level. A considerable reduction was observed in groups treated with* P. guajava* (100, 250 mg/kg) compared to glibenclamide group and thus reductions with other groups when compared with the diabetic untreated group.

**Figure 5:**
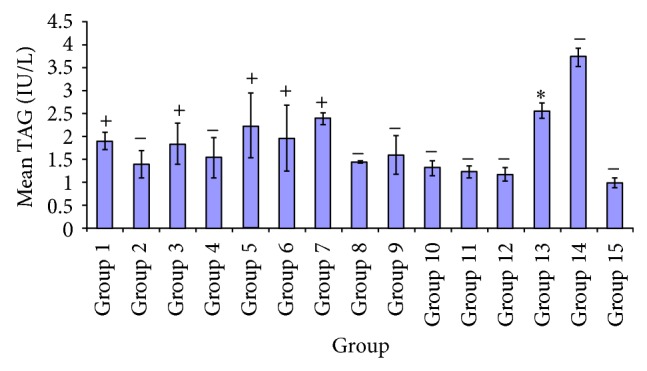
Comparative effect of the various plant extracts on triglycerides.

**Figure 6:**
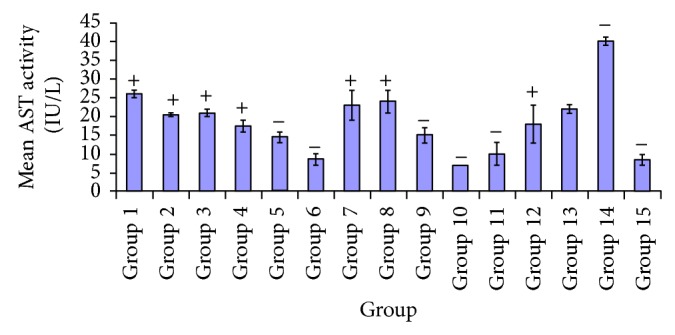
Comparative effects of the some plants extracts on aspartate aminotransferase (AST) activity.

In Figure 5, it can be deduced that there was a greater decrease in triglyceride level in groups administered with* A. occidentale *(250 mg/kg),* E. globulus* (250 mg/kg),* A. occidentale* +* E. globulus* (100, 250 mg/kg), and* P. guajava* +* X. aethiopica* (100, 250 mg/kg) and slight decrease with groups given* A. occidentale *(100 mg/kg),* X. aethiopica* (100 mg/kg),* P. guajava* (100, 250 mg/kg), and* X. aethiopica* (100 mg/kg) compared to the diabetic untreated group. Figure 6 shows the activity of aspartate aminotransferase based on the effect of the varying doses of the various plant extracts. From the plots it was deduced that there was more significant difference in AST levels of the groups administered* A. occidentale* +* E. globulus* (100, 250 mg/kg),* P. guajava* +* X. aethiopica* (100 mg/kg), and* P. guajava* (100 mg/kg) and a lesser reduction in groups* A. occidentale* (250 mg/kg),* E. globulus* (100, 250 mg/kg), and* P. guajava* +* X. aethiopica* when compared to glibenclamide group. Phytochemical screening of these plants showed the presences of alkaloids, flavonoids, tannins, saponins, and so forth, which are known to perform specific and various functions and hence exhibit different biochemical and pharmacological actions such as cell toxicity and cell protection [37].

The reductions in ALT and AST levels are said to be caused by the hepatocellular and cardiac protection offered by these extracts. This is further confirmed by the work of Shen et al., 2008, [38] polyherbal formulation in liver function enzymes in diabetic rats. That upon the administration of herbal formula led to a decrease in ALT and AST levels, as observed in groups treated with different doses of the polyherbal formulation which has* X. aethiopica* as one of its component. The implication of this is that the extract did not produce toxic effects on both the cardiac and hepatic tissues while in the diabetic untreated group there was a noticeable elevation in these two enzymes, an indication of hepatic and cardiac tissue damage. Components such as flavonoids a polyphenol have potent antioxidant capabilities. In 1986 Torell et al. [39] and Faure et al. [40] showed that they inhibited peroxidation of polyunsaturated fatty acids in cell membranes while [3] reported that flavonoids from* Helichrysum *genus inhibited the formation of two powerful peroxidation agents, namely, superoxide ions and hydroxyl radicals.

**Figure 7:**
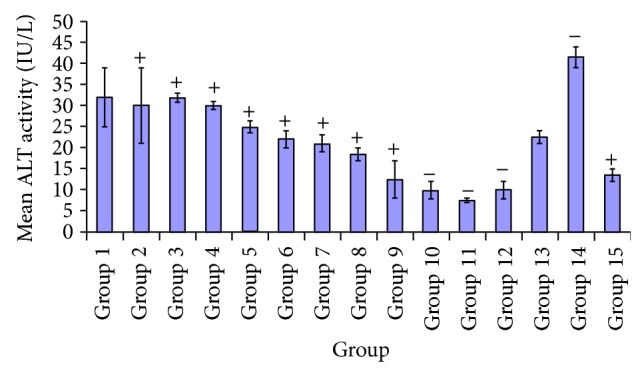
Comparative effects of some plants extracts on alanine aminotransferase (ALT) level.

However, Figure 7 shows the effect of the plant extracts on alanine aminotransferase (ALT) activity upon the administration of these extracts. A sharp reduction was observed in synergy groups administered* A. occidentale* +* X. aethiopica* (100, 250 mg/kg) and* P. guajava* +* X. aethiopica* (100, 250 mg/kg) compared to glibenclamide group. The groups treated with extracts* P. guajava* (250 mg/kg) and* X. aethiopica* (100, 250 mg/kg) had significant reductions in ALT levels compared to groups* A. occidentale* (100, 250 mg/kg) and* E. globulus* (100, 250 mg/kg) compared with the glibenclamide group.

**Figure 8:**
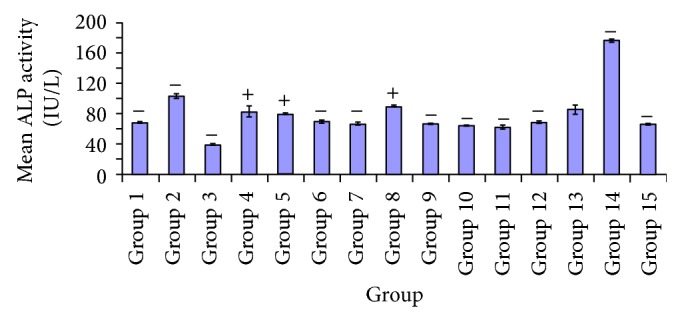
Comparative effects of different plants extracts on alkaline phosphatase (ALP) level.

**Figure 9:**
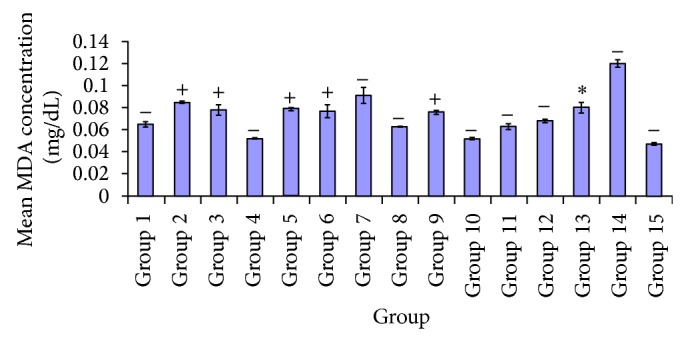
Comparative effect of the various plant extracts on malondialdehyde (MDA).

In Figure 8, the effect of varying doses of plant extracts on alkaline phosphatase activity in diabetic rats can be seen. A greater reduction is shown in groups administered* A. occidentale* (100 mg/kg),* P. guajava *(250 mg/kg),* E. globulus* (100 mg/kg),* X. aethiopica* (100 mg/kg),* A. occidentale* +* E. globulus* (100, 250 mg/kg), and* P. guajava* +* X. aethiopica* (100, 250 mg/kg). A minimal extent of reduction in the serum ALP levels in groups* E. globulus* (250 mg/kg) and* P. guajava* (100, 250 mg/kg) was observed compared to the glibenclamide treated group. A much more significant elevation plasma ALP levels were seen in diabetic untreated group compared to every other groups. The reductions in serum ALP levels are a result of cellular membrane/hepatocellular membrane protective effect of the plants extracts, which is supposedly a factor of phytochemical constituents. In 2010 Uboh et al. [41] showed that the aqueous extracts of* P. guajava* infer the same hepatocellular protection in rats that is linked to flavonoids, a phytochemical component. Flavonoids have been reported to possess antioxidant activity [42], thus protecting cell membranes from peroxidative actions of free radicals. Hence Figure 9 showed the effect of extracts on levels of malondialdehyde (MDA). A more significant decrease of MDA levels was observed in groups treated with* E. globulus* (250 mg/kg),* X. aethiopica* (250 mg/kg), and* A. occidentale* +* E. globulus* (250 mg/kg) and a significant reduction was observed in groups* A. occidentale* (100 mg/kg),* P. guajava* (250 mg/kg), and* P. guajava* +* X. aethiopica* (100, 250 mg/kg) compared to the glibenclamide and the diabetic untreated groups. These biochemical effects are supported by the works of Hsieh et al. [43] who reported that the polyphenolic components of* P. guajava* extracts have high concentration and its equivalent to gallic acid at the rate of 165.61 mgg^−1^. Also, polyphenolics and flavonoids are excellent scavengers of free radicals, ferrous ions chelators [44]. In 2007, Hsieh et al. showed that quercetin, a phytochemical component of gallic acid and ferulic acid in an extract of* P. guajava*, inhibited the formation of advance glycation and end-products. Quercetin has been shown in vivo and in vitro to possess antiglycative biochemical properties whereby it inhibits diabetic complications. Reference [45] also prevented oxidative *β*-cell damaged in streptozotocin animal subjects in vivo [46]. The work of [47] on the stem-bark extract of* A. occidentale* in rats fed with high-fructose diet did not cause plasma membrane damage.* Eucalyptus globulus* leaves contain flavonoids such as quercetin that possess antioxidant properties.

**Figure 10:**
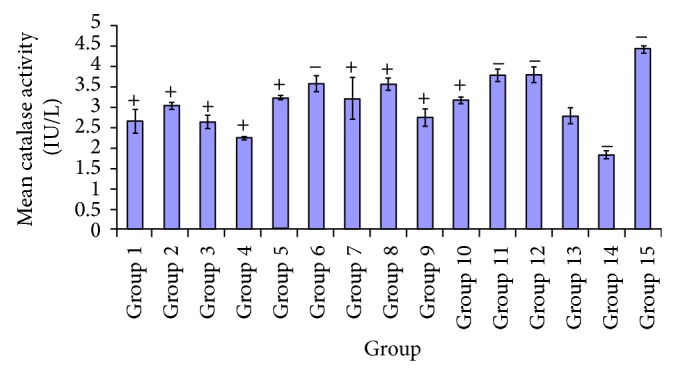
Comparative effects of some plants extracts on catalase level.

**Figure 11:**
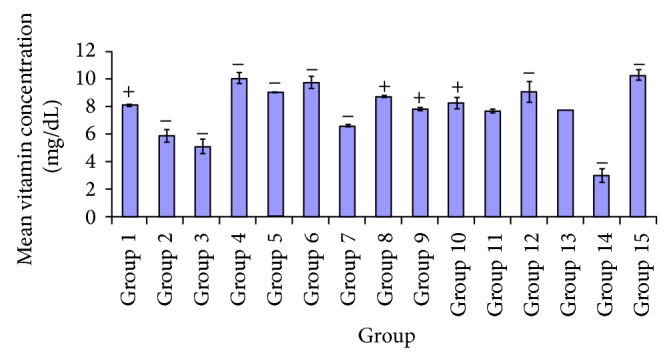
Comparative effect of the various plant extracts on vitamin C levels.

Figure 10 shows the effect of these plants extracts on catalase activity; from the plots a significant increase in levels of catalase was recorded in treated groups* P. guajava* (100, 250 mg/kg),* X. aethiopica* (100, 250 mg/kg), and* P. guajava* +* X. aethiopica* (100, 250 mg/kg). In Figure 11, the levels of vitamin C are depicted by the individual plots, as effects of the various extracts at varying doses with significant elevation recorded in groups* E. globulus* (250 mg/kg),* P. guajava *(100, 250 mg/kg),* X. aethiopica* (250 mg/kg), and* A. occidentale* +* X. aethiopica* (250 mg/kg), and fairly increase with groups treated with* A. occidentale* (250 mg/kg) and* P. guajava* +* X. aethiopica* (250 mg/kg). Also with a fair increase in groups administered* A. occidentale* (100 mg/kg) and* A. occidentale* +* E. globulus* (250 mg/kg), compared to the glibenclamide treated. The elevations in the levels of these enzymic and nonenzymic antioxidants after the administration of the various plants extracts indicate the presence of antioxidant components that offers cellular protection from peroxidative actions of free radicals produced during diabetes mellitus. From the various figures and plots of these antioxidants with regard to the effect of the extracts, it can be inferred that the antioxidant levels in the diabetic untreated groups show significant reductions. This implies that peroxidative reactions by free radicals had occurred. Therefor, the marker for this biochemical process “malondialdehyde” is a factor for the determination of this reaction.

**Figure 12:**
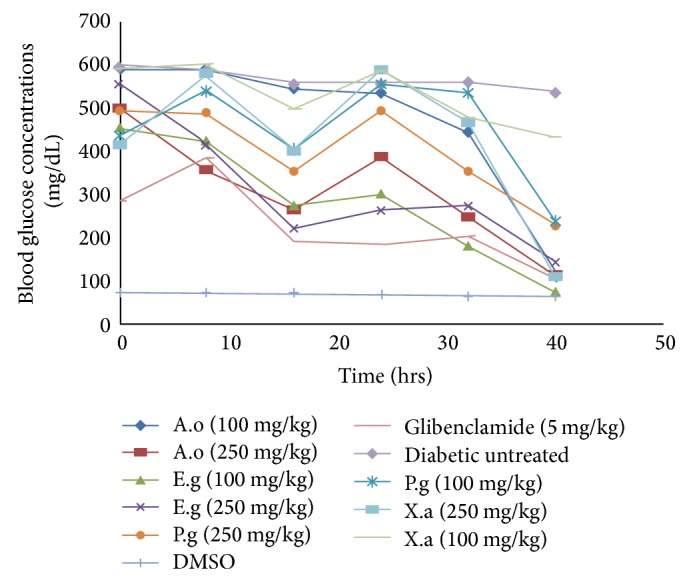
The effect the various plant extracts on blood glucose concentrations at different time intervals. A.o =* Anacardium occidentale*, E.g =* Eucalyptus globulus*, P.g =* Psidium guajava*, and X.a =* Xylopia aethiopica*.

Results of Figure 12 show the effect of plants extracts on blood glucose concentration, which records glucose level relative to time and dose as seen in the trend of decline. Sokeng et al. [48] showed that the methanolic extract of* Anacardium occidentale* effectively reduced the blood glucose level in streptozotocin-diabetic rats; this reduction was shown to be more effective with subsequent fractions of the methanolic extract. The mechanism of these extracts supposedly is attributed to the direct stimulation of insulin in the remaining pancreatic cells. In other words the extracts are involved in an insulin-like extrapancreatic stimulation, such as the stimulation of glucose utilization and reduction of hepatic gluconeogenesis [49]. Repeated administration of methanolic extracts and its fractions culminated in a decrease in blood and urine glucose levels [48]. The hypoglycemic effects of these plants are further attributed to their phytochemical constituent. Soussi et al. [50] examined the antihyperglycemic/hypoglycemic effect of* Eucalyptus globulus* in diabetic mice, whereby dietary administration of these plants ameliorated loss of body weight, polydipsia, and blood glucose level in streptozotocin-diabetic mice. This effect is linked to the protection or regeneration of pancreatic *β*-cell following the exposure to the diabetogen-streptozotocin and its action by modulation of insulin secretion. Also in the works of Soussi et al. [50] the pancreatic effects of these plants and their blood glucose lowering ability were reported. The antidiabetic effect of* Psidium guajava* leaves is connected to their phytochemical constituent such as the various flavonoids, terpenoids, and glycosides as reported by [7, 51, 52]. The leaves of these plants are used in the reduction of postprandial blood glucose elevation, improvement of hyperinsulinemia in marine models [53]. Also, Pinto et al. [54] in his experiment tested a group of diabetic rats by the administration of some herbal plants that had* P. guajava* as one of them. Upon the oral administration of aqueous extracts of the leaves at a dose 500 mg/kg body weight for 15 days on, recorded a significant reduction of blood glucose as 43.59% compared to 47 and 74% of glibenclamide.

**Figure 13:**
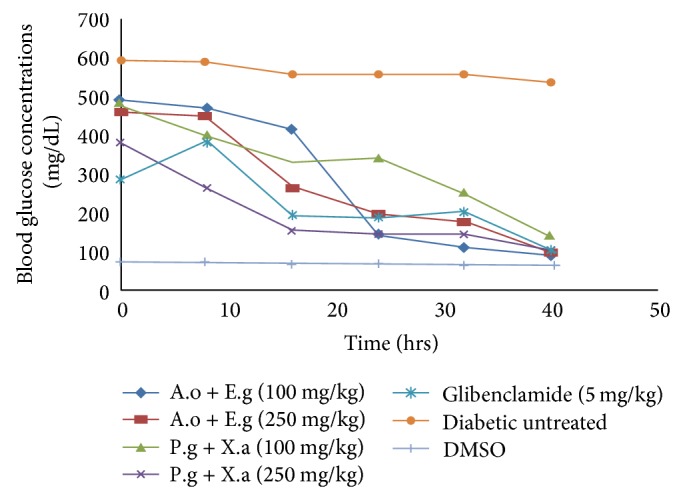
The effect of combined plants extracts on blood glucose concentrations at different time intervals. A.o + E.g =* Anacardium occidentale* +* Eucalyptus globulus*, P.g + X.a =* Psidium guajava* +* Xylopia aethiopica.*

The fruits of* Xylopia aethiopica* have been reported to possess hypoglycemia ability as well as other biochemical activities, thus confirming its usage as an antidiabetic agent [55]. This was further supported by the polyherbal formula of Shen et al., [38] which composed of* X. aethiopica *as one of its component, which showed a significant reduction in plasma blood glucose upon the administration of alcoholic extracts of polyherbal formula. This was found to have done better than glibenclamide. Also, in 2008 Shen et al. showed a significant decrease in plasma glucose of diabetic Wister rats upon the administration/treatment with plant extracts where* X. aethiopica* was one of the extracts. The reduction in blood glucose was found to be dose dependent. Figure 13 showed greater reduction in plasma blood glucose with synergic formulations of extracts* A. occidentale + E. globulus* compared to that of* P. guajava* +* X. aethiopica* and glibenclamide. This is largely attributed to the plants extracts combined formulation, which is gotten from the individual hypoglycemic activity shown in Figure 13. This is further buttressed by the synergetic works of Shen et al., 2008, on the polyherbal formulation that showed effective reductions in plasma glucose concentrations of diabetic rats than that of glibenclamide. A factor responsible for this could be the synergistic interaction of some phytochemical components such as polyphenols and tannins.

Glucose-6-phosphatase is a key enzyme in the homeostasis of blood glucose through the catalysis of terminal step both in gluconeogenesis and glycogenolysis. Fructose-1, 6-bisphosphatase is one of the key enzymes of gluconeogenic pathway [57].

Hepatic, cardiac tissues release aspartate aminotransferase and alanine aminotransferase; therefore the elevation of plasma concentrations of these enzymes is an indicator of hepatic and cardiac damage [58], as in the case of complications in diabetes mellitus while alkaline phosphatase function as a biochemical marker enzyme for maintaining membrane integrity [59]. The increase in plasma levels of this enzyme suggests peroxidation of cell membrane, which occurs during diabetes.

## 10. Conclusion 

These individual plants extracts exhibited significant increase in ameliorating diabetes complication and blood glucose control compared to glibenclamide, a synthetic antidiabetic drug. Greater performance was observed in the synergy groups. Therefore, a poly/combined formulation of these plants extracts will give considerable effects as well as yielding significant result and may resolved some other complications associated with diabetics.

## Figures and Tables

**Table 1:** 

	Mass	Extraction yield
*Anacardium occidentale *	73.0 g	12.2%
*Eucalyptus globulus *	137.0 g	22.8%
*Psidium guajava *	90.0 g	15.0%
*Xylopia aethiopica *	35.0 g	5.8%
*A*. *aethiopica + E*. *globulus *	110.0 g	18.3%
*P*. *guajava + X*. *aethiopica *	105.0 g	17.5%
